# Field-based determination of controls on runoff and fine sediment generation from lowland grazing livestock fields

**DOI:** 10.1016/j.jenvman.2019.109365

**Published:** 2019-11-01

**Authors:** S. Pulley, A.L. Collins

**Affiliations:** Sustainable Agriculture Sciences, Rothamsted Research, North Wyke, Okehampton, Devon, EX20 2SB, UK

**Keywords:** Erosion, Grassland, Farming, Lowland grazing, Rain splash, Saturation-excess runoff

## Abstract

Compared with arable land, there is a paucity of field-based measurements of erosion rates and controls for lowland temperate grassland supporting ruminant agriculture. Despite this evidence gap, reducing diffuse fine sediment pollution from intensively farmed grassland has been recognised as essential for improving compliance with water quality targets. Improved information on erosion rates and controls within intensively managed lowland grazing livestock systems are prerequisites for informing best management practices for soil and water resource conservation.

Accordingly, this study assembled such information using the North Wyke farm platform in south west England where flow, suspended sediment concentration, rainfall and soil moisture are monitored quasi-continuously in 15 hydrologically-isolated (1.54–11.12 ha) catchments. This region of the UK is representative of temperate lowland ruminant grazing landscapes with semi permeable soil drainage.

Catchment area was the major control on both water and sediment flux. When normalised to catchment area, sediment yields were controlled by the erodibility of the catchment's soils. Ploughing for re-seeding of grass swards was the major factor that affected this. Whilst total rainfall had a small effect on sediment yields, slope and the damage of soils by livestock had no significant effects. This finding may be due to the overriding effects of ploughing and re-seeding of some fields during the study period.

Detachment by impacting raindrops mobilised sediment particles across the entire field with diffuse saturation-excess overland flow responsible for their transport. The majority of erosion occurred during the rising limbs of storm events when there is an abundance of easily detached soil particles. Given that erosion and sediment transport are driven mechanistically by processes affecting the entire field areas, a reduction in sediment yield through the implementation of highly spatially-targeted in-field management such as that for feeder ring use, troughs, poached tracks or gateways would likely be very challenging. Instead, stocking density and grazing regime management, as well as carefully planned ploughing and re-seeding will be more beneficial for erosion control.

## Highlights

• Water and sediment fluxes were strongly correlated with catchment area.
• Sediment yields significantly increased after ploughing.
• Rainsplash detached particles whilst saturation-excess overland flow transported them.
• No significant effect of slope or soil poached by livestock on sediment generation was found.
• The entire areas of each catchment are likely to be contributing sediment to the flumes.

## 1 Introduction

Soil erosion and the resulting diffuse fine sediment pollution from agriculture has been identified as a leading cause of the degradation of freshwater habitats (Berkman and Rabeni, 1987; Wood and Armitage, 1997; Kemp et al., 2011). Diffuse agricultural sediment has resulted in significant off-farm costs; for example, Collins and Zhang (2016) calculated that maximum environmental damage costs of £523 M yr^−1^ are incurred in the UK due to detrimental effects of agriculturally-related sediment pollution on ecosystem goods and services. To mitigate these effects and achieve compliance with water quality targets it has been estimated that sediment loads from agriculture will have to be reduced by up to 20% (Collins and Anthony, 2008). Such an achievement may be challenging, however, since Zhang et al. (2017a) recently estimated that best-case future on-farm management interventions costing a median of £13,000 km^−2^ yr^−1^ are only likely to deliver a median 25% reduction in agricultural sediment loss under business-as-usual. In the context of this management challenge, it is necessary to target on-farm mitigation on the basis of robust data on pollution losses and key controls to deliver optimum cost-benefit (Ockenden et al., 2012). Erosion rates and controlling processes must therefore be understood in a range of different agricultural landscapes. Accordingly, this study focuses on assessing the magnitude of sediment loss from intensively managed lowland grassland in a UK setting. Grasslands represent a significant proportion of land internationally. Nationally in the UK, grassland represents 67% of the total agricultural land area (Defra, 2015), whereas it represents ~40% of the agricultural area of western Europe (Peeters, 2004), and grassland/range land also represents 35% of land area in the USA (Environmental Protection Agency, 2019).

Soil erosion on grasslands is generally less severe than on cultivated land due to its near continuous vegetation cover (O'Connor, 1956; Patto et al., 1978; Bilotta et al., 2007a; Evans, 2010), and, as a result, until recent decades, most monitoring studies in the UK have focussed on assembling data on erosion rates and key controls in arable settings (Evans, 1988, 1990a; Boardman et al., 2009; Boardman, 2013, 2015). However, a decline in aquatic habitat quality and non-compliance with water quality targets has been attributed to agriculturally-derived sediment pollution in grassland dominated catchments (Heathwaite et al., 1990; Hooda et al., 2001; Preedy et al., 2001; Harrod and Theurer, 2002). Whilst the soil erosion rates and sediment yields in UK catchments which average approximately 44 t. km^−2^ yr^−1^ (Walling et al., 2008), are low compared to other regions of the world (Tilahun et al., 2015), and do not pose a threat to soil as a resource in the majority of areas, there are significant costs associated with remediating the off-site impacts of diffuse pollution by sediment (Collins and Zhang, 2016; Zhang et al., 2017a). In addition, fine sediment emitted from grasslands acts as a vector for particulate phosphorous delivery to freshwater receptors (Heathwaite and Dils, 2000; Hooda et al., 2001; Granger et al., 2007) underscoring the wider environmental significance of elevated soil erosion and sediment loss under modern intensive farming practices.

Multiple factors have been suggested to explain erosion rates on temperate grassland fields. For example, high stocking densities associated with intensive lowland livestock farming can cause the compaction, poaching and pugging of soils (Foster and Walling, 1994; Evans, 1998; Harrod and Theurer, 2002; Bilotta et al., 2007b). Such disruption of soil structure can reduce soil infiltration capacity and increase surface runoff volumes and associated erosion risk (Heathwaite et al., 1990). Increased soil moisture content has also been shown to increase soil damage by animals (Wind and Schothorst, 1964; Climo and Richardson, 1984; Scholefield and Hall, 1986), and can also reduce the internal friction between soil particles reducing resistance to poaching damage and erosion (Patto et al., 1978). In addition, the loss of vegetation associated with intensive grazing can expose bare soil to erosion (Thorsteinsson et al., 1971; Evans, 1977, 1997). The growing trend in the outdoor-wintering of livestock in the many temperate landscapes is likely to compound many of these factors. Slope has also been shown to be a major control on erosion rate (Ekern, 1953; De Ploey and Savat, 1968), which is linked with the fact that erosion has been shown to be directly related to runoff velocity (Moss, 1988; Kinnell, 1990a, 1990b). Field topography has also been linked to increased erosion with saturation-excess overland flow developing in the topographic convergence of valley axes (Dunne and Black, 1970; Anderson and Burt, 1978).

Many studies into soil erosion rates and key controls have historically been conducted at plot scale (Mutchler et al., 1994). It is questionable, however, if such results can be reliably upscaled to field or catchment scale (Evans, 1988; Lane et al., 1995; Seyfried and Wilcox, 1995), despite these scales being fundamental units for land management by farmers and for water quality compliance reporting in the context of policy-driven environmental objectives. Previous UK-based field scale studies into soil erosion rates have focussed on measuring visible rill and gully erosion and estimating sheet wash on cultivated land rather than assembling data for intensively managed grasslands (Evans, 1988; Boardman, 2003). It has also been identified that the prediction of erosion rates and processes through modelling is more challenging for smaller catchments when compared to large catchments (De Vente et al., (2013). Therefore, there remains a paucity of field scale information on soil erosion and its major controls within grassland landscapes. In this context, the aim of this study was to determine how and when sediment is transported from intensively managed grassland fields in a temperate lowland agricultural landscape with semi-permeable soil drainage. More specifically, the work determined which geographical and hydrological factors control the sediment loads and yields originating from 15 field scale catchments on the North Wyke farm platform (NWFP) in south west England.

## 2 Study site

**Fig. 1:**
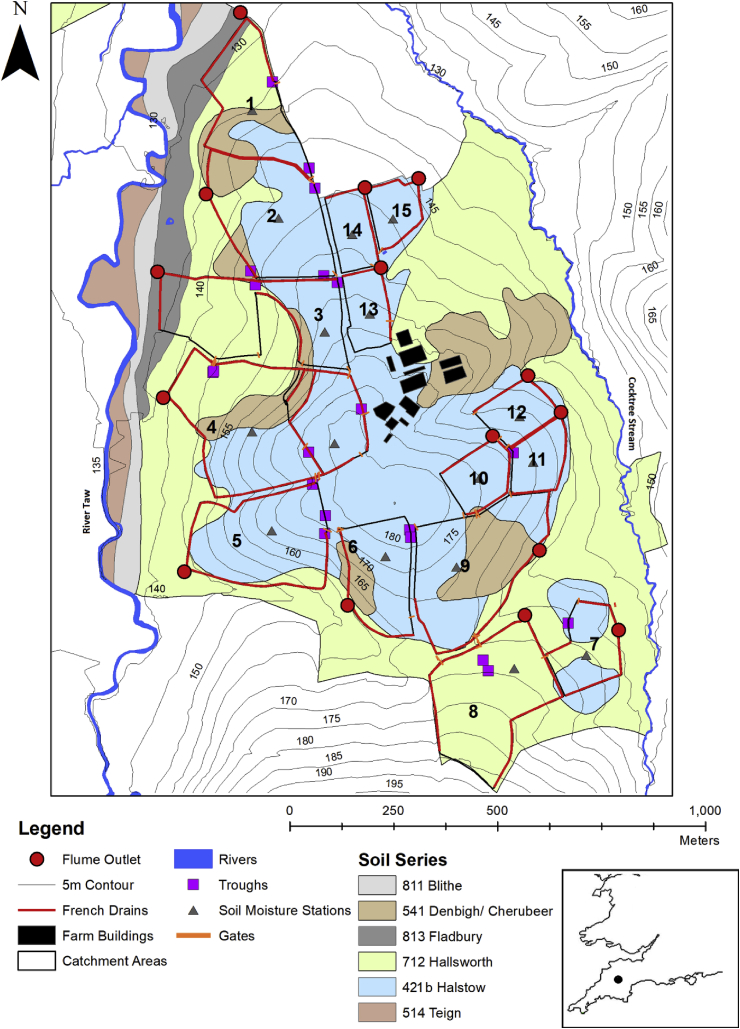
The North Wyke farm platform; each hydrologically-isolated catchment is marked and labelled with its flume number to correspond with the background information in Table 1.

The NWFP (50°46′10″ N, 30°54′05″ W) was set up in 2010 and is designed to test the productivity and environmental sustainability of temperate grassland beef cattle and sheep systems using three different swards; long-term permanent pasture, increasing use of legumes, and planned re-seeding with novel high sugar grasses. The platform consists of 15 hydrologically-isolated catchments draining either one or two fields which are monitored quasi-continuously for water, sediment and nutrient exports (Fig. 1). Full details of the design of the platform and the full range of monitoring equipment are provided in Orr et al. (2016).

The NWFP is located on raised ground between the River Taw and its tributary Cocktree stream. The hydrological areas of the catchments bounded using a network of French drains (800-mm deep trenches containing a perforated drainage pipe backfilled to the surface with 20–50 mm clean granite, carbonate free, stone chips) range from 1.54 ha for Flume 15 to 11.12 (subsequently reduced to 7.75 on 13/08/2013) ha for Flume 4 (Table 1). Many of the catchments are steeply sloping with the highest individual mean (Catchment 5, Table 1) being 12.25°; however, some catchments have a mean slope as low as 4.17° (Catchment 14, Table 1). The fields generally have a slowly permeable slightly stony clay loam topsoil (~36% clay) prone to seasonal waterlogging, which overlies a mottled stony clayey poorly permeable subsoil (~60% clay) derived from the underlying Carboniferous Culm rocks (Harrod and Hogan, 2008). The dominant soils (Fig. 1) have been classified as belonging to two similar series (Avery, 1980); Hallsworth (712, (Dystric Gleysol) and Halstow (421 b, Gleyic Cambisol). These intrinsic soil profiles provide the basis for the interception of lateral runoff and associated suspended sediment by the French drains inserted along the boundary of each catchment (Fig. 1). Only Flume 2 shows any evidence of rill erosion where a spring reaches the surface and produces a short area of rilling in the centre of the catchment, but this does not extend to the field margins. Bare and trampled soil is often present along field margins and around feeding troughs in all catchments where livestock are present. There is no gullying present within the study landscape.

**Table 1:** Characteristics of the 15 NWFP hydrologically-isolated catchments.

Flume number	Name	Area (ha)	Mean slope (degrees)	Percentage time animals present		Maximum number of animals present		Percentage of soil area				Ploughed
				Cattle	Sheep	Cattle	Sheep	421 b	712	541	813	
1	Pecketsford	4.81	5.83	13.27	32.28	32	103	22.8	41.4	25.5	10.3	No
2	Great Field	6.65	6.08	8.21	0	32	0	68	14.1	17.9	0	Yes
3	Poor Field	6.62	7.29	31.87	23.53	30	98	34.1	49.3	6	10.6	No
4	Burrows^a^	7.75 (11.12)	10.76	5.2	14.64	30	99	57	27	16	0	No
5	Orchard Dean	6.54	12.25	19.43	2.6	32	29	85	15	0	0	No
6	Golden Rove	3.86	9.76	0	27.77	0	106	74.9	5.1	19.9	0	No
7	Lower Wyke Moor	2.6	7.54	0	36.53	0	101	57.5	42.5	0	0	No
8	Higher and Middle Wyke Moor	7.02	6.77	5.2	2.87	32	92	0.6	99.4	0	0	Yes
9	Dairy Corner	7.75	8.42	39.95	20.38	32	74	56.9	5.8	37.3	0	No
10	Lower Wheaty	1.82	7.24	0	16.83	0	83	98.1	0	1.9	0	No
11	Dairy East	1.76	9.71	0	15.6	0	81	99.3	0.7	0	0	No
12	Dairy North	1.78	10.69	0	18.33	0	83	100	0	0	0	No
13	Longlands South	1.75	7.24	0	16.96	0	50	100	0	0	0	No
14	Longlands North	1.72	4.17	0	18.47	0	49	100	0	0	0	Yes
15	Longlands East	1.54	5.32	0	17.37	0	49	100	0	0	0	Yes

712, Hallsworth, Slowly permeable clayey soils often over shale. Some well drained fine loamy soils.

421 b, Halstow, Slowly permeable clayey soils often over shale. Some well drained fine loamy soils.

541, Denbigh. Free draining permeable soils on hard (slate and shale) substrates with relatively low permeability and low storage capacity and Crediton Free draining permeable soils on soft sandstone substrates with relatively high permeability and high storage capacity.

813, Fladbury, Stoneless clayey soils, in places calcareous variably affected by groundwater. Flat land Risk of flooding.

a The area of Flume 4 was reduced to 7.75 ha on the 13/08/2013.

This study focussed on a time period (1/10/2012–31/05/2014) encapsulating both baseline conditions when all fields were managed under long-term permanent pasture and a transition period of ploughing and re-seeding in the fields draining to Flumes 2,8,14 and 15. The study period therefore captured and provided a basis for comparing and contrasting sediment loss under the two extremes of land cover within the grazed systems; well-developed permanent pasture and bare ploughed re-seeded soil giving rise to a new grass sward in line with the overarching experimental objectives of the NWFP to test the production and sustainability of beef and sheep farming with different swards. Beef cattle were only present in catchments 1, 2, 3, 4, 5, 8 and 9 during the study period, whereas sheep and lambs were present in all catchments apart from 8. Where cattle were present between 13 and 32 individuals were in each catchment; this number, however, varied considerably over time. Up to 103 sheep and lambs were present in the catchments and like the cattle the number present varied significantly over time. Management in the non-grazed catchments involved mowing and two silage cuts each year. Fertiliser was also applied to the catchments. Feeder rings are moved regularly in tandem with policy guidance and troughs generally have concrete bases. Average annual rainfall during the study was 1252 mm which is higher than the long-term average of 1053 mm.

## 3 Material and methods

### 3.1 Data collection

The drains from each catchment on the NWFP converge on a pre-collection chamber where samples of the runoff are extracted for measuring pollutant (e.g. sediment) content. Runoff then passes to an open channel where flow is recorded at 15-min intervals using H-flumes (designed for a 1 in 50-year runoff event) equipped with Tracom flow height gauges, and Teledyne ISCO bubbler flow-meter devices (ISCO Open channel flow measurement handbook, 2001). Turbidity is also measured at 15-min intervals using YSI multiparameter sondes (6600V2, YSI) inserted into stainless steel bypass flow cells. The latter are necessary to ensure that the multi-parameter sondes do not become vulnerable to drying out during the absence of field runoff. The sondes are calibrated quarterly for turbidity measurements using a two-point calibration; 0 (RO water) and 124 formazine nephelometric turbidity units (FNU). Automatic water samplers (ISCO) are used for the routine collection of water samples for developing the suspended sediment concentration-turbidity ratings for the turbidity sensors.

Recorded turbidity was converted into suspended sediment concentration (SSC) using ratings developed using 100 ml samples of runoff from the flumes sampled over a range of flow conditions. These samples were filtered through 0.7  μm pore size glass fibre paper and oven dried at 105 °C for 60 min (Equation (1); Bilotta et al., 2008). For Flumes 2, 8, 14 and 15 it was found that the relationship between SSC and turbidity changed after ploughing in mid-2013. As such, a new rating (Equation (2)) was developed from the relationship shown in Supplementary Fig. 1 and this was applied during the post-plough period, defined as the time spanning from ploughing till the end of the first winter (March 31st) post plough.

(1) $$SSC=1.1804⁎NTU+0.0472\left(r^{2}=0.75\right)$$

(2) $$SSC=0.7664⁎NTU+5.7116\left(r^{2}=0.91\right)$$

It was not possible to obtain a measurement of turbidity during flows of less than 0.0002 m^3^ s^−1^ due to inadequate water depth; the intercept value of the SSC-turbidity relationships was therefore used for these periods in the runoff records.

An Adcon SM1 soil moisture station and an ADCON RG1 tipping bucket rain gauge with 0.2 mm resolution have been installed in the centre of each catchment. The soil moisture station records percentage moisture using capacitance at depths of 10, 20 and 30 cm. Both soil moisture and rainfall are recorded at 15-min intervals.

The particle size distributions of the <2 mm fraction of soils within each catchment were quantified using a composite sample of 10–33 individual topsoil samples collected to a depth of 10 cm using a steel auger and a grid sampling strategy. Each composite sample was wet sieved through a series of stainless steel meshes (1000, 500, 250, 125, 63, 45 and 25 μm) and the percentage of the total sample mass retained in each sieve was recorded.

### 3.2 Data analysis

Data analysis comprised three stages. In stage one, the temporal trends in sediment and water flux were explored. Here, the percentage of the total sediment and water flux which occurred under each percentile of total flow rate in 5th percentile increments (0–5th, 5th-10th, etc) was determined to identify under what conditions the majority of export occurred. A time series of high flow events in Flume 4 (the largest catchment) was then examined to compare the temporal relationships between rainfall and sediment flux. The time series for the entire study period was also examined for the catchment which had the most complete soil moisture dataset (Flume 10) to determine the effect of soil moisture (%) on water and sediment fluxes. As part of this analysis, the relationships between flow and SSC were examined during saturated and non-saturated soil conditions. Finally, the relationships between flow and SSC were examined both before and after ploughing in Flume 8 which had the highest overall sediment export.

In stage 2, fluxes of water and sediment were examined to determine their controlling factors. The differences between total rainfall in each flume were first compared to determine how spatially variable the quantities of water delivered to the catchments were. It was also determined what percentage of the rainfall delivered to each catchment reached the corresponding flume outlet. The data time series was then divided into three categories based upon flow condition; rising limb, falling limb and baseflow. Where there was an increase in flow from the previous measurement the 15-min time period was classified as the rising limb, where there was a decrease it was classified as the falling limb, and where flow was at a rate less than 5% of the maximum value recorded in each flume the time period was classified as under baseflow conditions. This threshold was determined through a visual inspection of the flow time series; the 5% threshold was judged to best separate the rapidly rising or falling high flow peaks from flat or gently falling flow rates which characterised periods with little rainfall. The flow condition category was only changed from the previous if it lasted for longer than 1 h to minimise the noise of short duration fluctuations in flow. The total water and sediment fluxes were calculated for each flow condition as well as the total for the entire study period. It was then determined what percentage of the water and sediment fluxes and percentage of the study period duration occurred under each flow condition. The sediment and water export for each flume were compared to catchment characteristics (described at the end of this section) and the mean SSC of the flume runoff to determine their controlling factors.

In the final part of the analysis, the water and sediment fluxes were normalised to catchment area and the total duration of each flow condition to generate water and sediment specific yields (m^3^ ha^−1^ yr^−1^; t ha^−1^ yr^−1^). The yields were compared to catchment characteristics and the mean SSCs in the runoff of each flume to determine their controlling factors. A Pearson correlation matrix was finally used to summarise all of the correlations between catchment variables and sediment and water fluxes and yields.

The catchment characteristics compared to sediment and water fluxes and yields comprised: rainfall (mm), measured by the tipping bucket rain gauges; the percentage of time livestock were present, determined through farm management records; the percentage of soil area damaged by livestock and the total area of damaged soil (m^2^), identified manually using an 5 cm resolution aerial photograph and NDVI in ARCGIS 10.5; rainfall reaching each catchment outlet (%), calculated by multiplying the total rainfall by the catchment area and dividing by the measured water flux at the corresponding flume; catchment area (ha); mean catchment slope (°), measured using a 5m resolution Ordinance Survey Terrain 5 DEM in ARCGIS 10.5; rising limb water flux (m^3^), falling limb water flux (m^3^) and baseflow water flux (m^3^), calculated using the flume measurements; maximum flow accumulation in each catchment (m^3^), calculated using a 5m resolution DEM and the flow accumulation tool in ARCGIS 10.5; total water flux (m^3^); percentage of time spent in the rising limbs of all storm events across the study period; percentage of time spent in the falling limbs of all storm events across the study period.

## 4 Results

### 4.1 Temporal trends in runoff and sediment generation

**Fig. 2:**
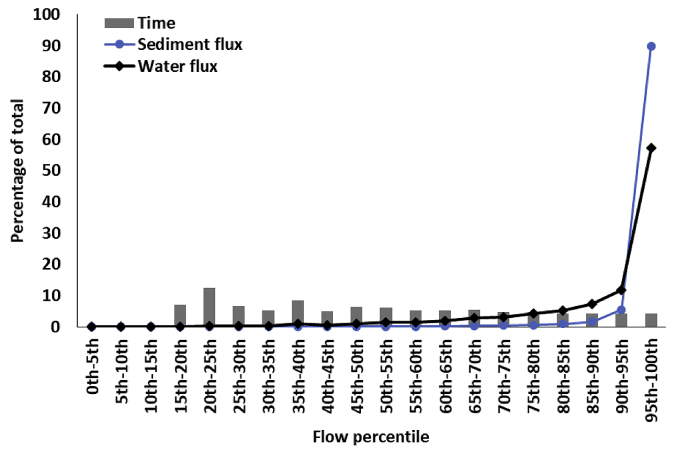
The percentage of total water and sediment flux taking place under each percentile flow rate in Flume 4.

In almost all flumes, over half of the water flux took place between the 90th and 100th percentile flow rate despite this covering only 4% of the monitoring period duration (see example using Flume 4 in Fig. 2). In excess of 90% of the total sediment flux took place during the top 5th percentile of flow conditions, indicating that the short periods of very high flow dominate sediment fluxes from the catchments (see example using Flume 4 in Fig. 2). The proportion of the study period where sediment was transported in concentrations exceeding background was highly variable between the flumes. Flumes 1, 3, 4, 5 and 9 generated sediment during more than 50% of the study period. In contrast, Flumes 12 and 13 only generated sediment for 20% of time, whilst Flumes 2, 6, 7, 8, 10, 11 and14 generated sediment for between 20 and 30% of the study period. There was a significant positive relationship between the total percentage of the study period duration spent in a high flow event and catchment area, with an r^2^ of 0.55 (Supplementary Fig. 2a). Flume 15 was the exception to this relationship, with long duration flows for its area; when this outlier was removed, the r^2^ increased to 0.87.

**Fig. 3:**
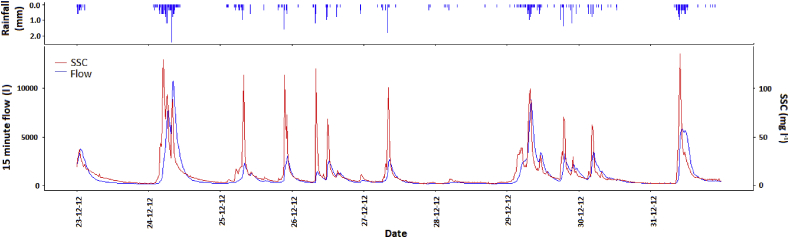
Flow, rainfall and SSC time series for Flume 4 from the 23/12/2012 to the 31/12/2012.

It was observed that a rise in turbidity occurred during periods of active rainfall and generally dropped sharply after rainfall stopped, even when flow remained elevated (see example using Flume 4 in Fig. 3). This trend was present for most large storm events in most flumes and was most pronounced in the flumes with the highest SSCs in an event. Further rainfall during the same high flow event often resulted in turbidity rising again. For example, the event on the 24/12/2012 (Fig. 3) experienced an hour of heavy rainfall after the initial peak in SSC had fallen which resulted in a second peak in SSC. Fig. 3 demonstrates that the rise in SSC occurs prior to the rise in flow suggesting that sediment mobilisation and transport can occur with only small quantities of overland flow. This pattern was common to most catchments where a high SSC occurred (see Supplementary Fig. 3).

**Fig. 4:**
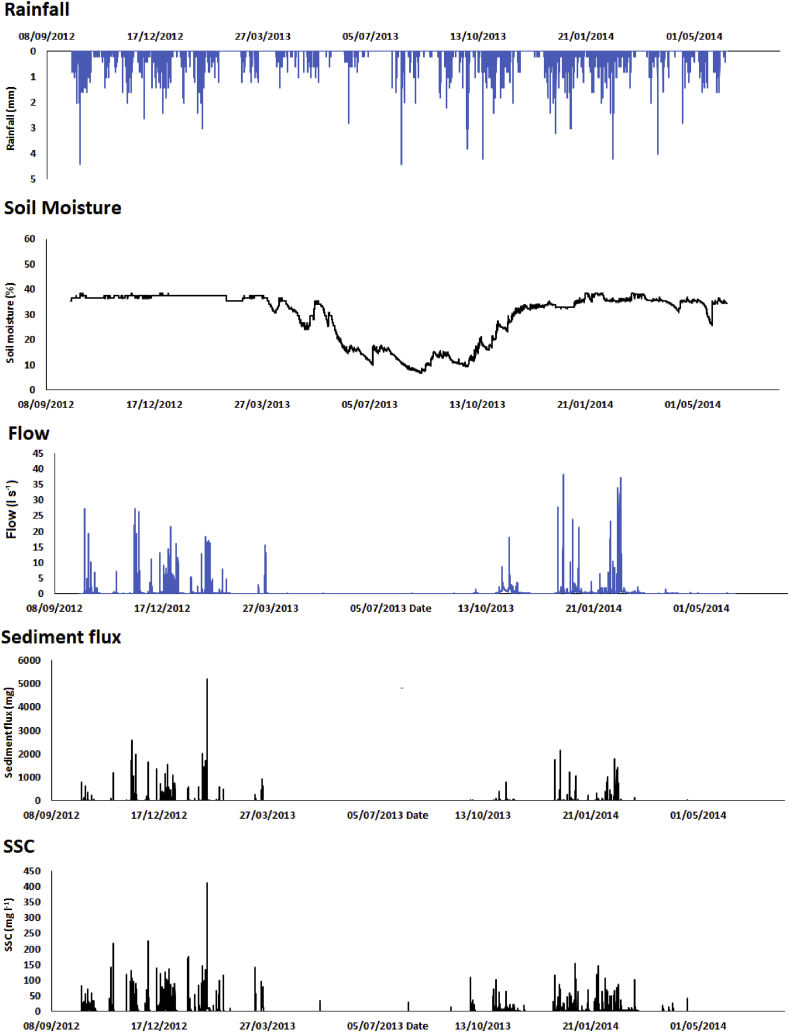
Rainfall, soil moisture, flow, sediment flux and SSC time series for Flume 10.

**Fig. 5:**
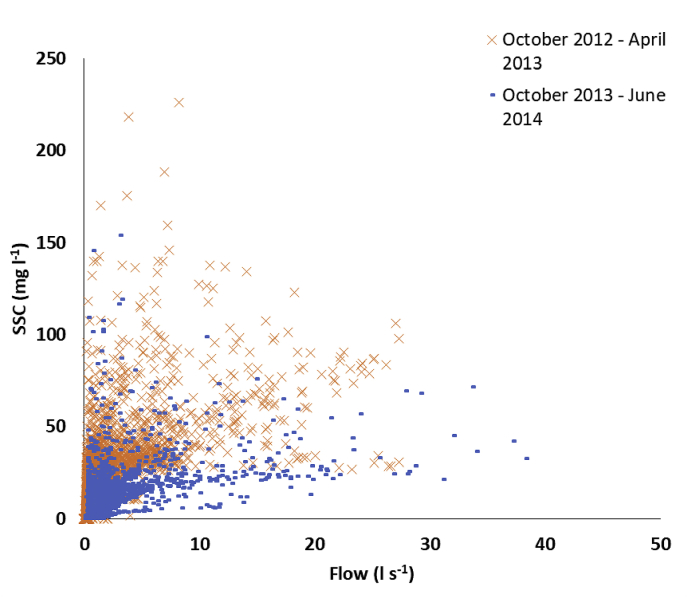
Relationships between flow and SSC during winter – spring 2012–2013 and 2013–2014.

All flumes were characterised by a reduction in soil moisture during the summer of 2013 due to a period of low rainfall and high evapotranspiration (Fig. 4). During this dry period large rainfall events did not result in any appreciable flows or sediment generation. The high rainfall of mid-October 2013 increased soil moisture but it remained lower than that during the previous winter until the end of the study period. This had a large effect on the capacity of the catchments to generate sediment. After October 2013, there was a far lower SSC for a given flow in each flume than prior to the reduction in soil moisture in April 2013 (Fig. 5).

**Fig. 6:**
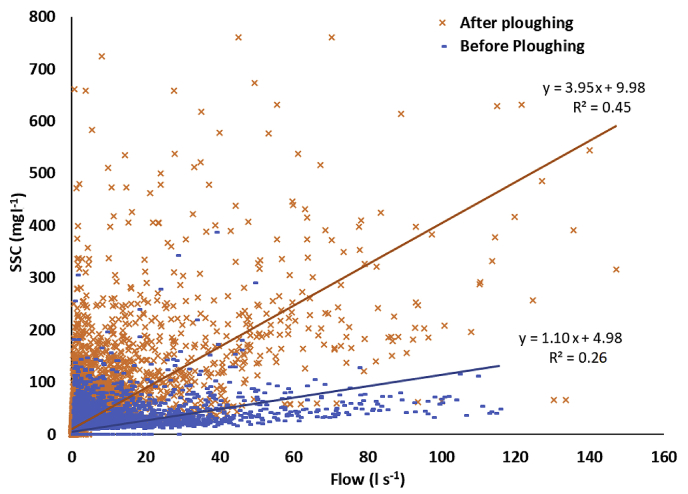
The relationship between SSC and flow before and after ploughing in Flume 8.

The ploughing and re-seeding of the fields draining to Flumes 8, 14 and 15 caused a significant increase in the mean SSC for a given flow rate in the post-plough period. This increase was most pronounced in Flume 8, where SSC for a given flow rate could be over 7 times higher than before ploughing despite soil moisture content being lower during this transition period (Fig. 6; plots for the other ploughed catchments are provided in Supplementary Fig. 4). Ongoing work is exploring explanations for the differing responses to ploughing. The SSC for a given flow rate increased under all flow conditions but was most significant during higher flows.

### 4.2 Fluxes of water and sediment

#### 4.2.1 Temporal patterns of flow

When the flow time series were divided into the rising limb, falling limb and baseflow for all flumes, baseflow conditions (<5% of maximum flow) were present for the vast majority of the study period. In only the largest catchment (Flume 4) was less than 90% of the monitoring period spent under baseflow conditions. For all flumes, the mean percentage of time spent in the rising limb and falling limbs were comparable at 2.21% and 2.63%, respectively. There was, however, some variation between flumes with, for example, 6.97% of time spent in the rising limb for Flume 15 and only 0.25% for Flume 12 (Supplementary Table 1).

Mean precipitation during the study period for all flumes was 2079 mm (Table 2). Flumes 7, 8 and 11 had the highest rainfall with 99–168 mm more than the mean whereas Flumes 5 and 6 had the lowest at 182 and 160 mm less than the mean, respectively. There is a general spatial trend of the south east catchments experiencing the most rainfall and the south west the least. This spatial pattern is likely to reflect the orographic effect of winds from the south west having to transverse more of the neighbouring Dartmoor upland than winds from the south east. When the total rainfall inputs to each flume were compared to the monitored total water yields of the corresponding catchments (Table 2), Flumes 1 and 15 had the highest proportion of rainfall delivered to the flume outlet at 72% and 77%, respectively, compared to an overall mean of 53% for all flumes. Rainfall intensity when quantified using a histogram of daily rainfall totals is related to total rainfall with Flumes 4, 5 and 6 in the south west having 44, 44 and 52 days with rainfall exceeding 10 mm, compared with a mean of 62.5 days for the other flumes (Supplementary Fig. 5).

**Table 2:** The total rainfall in each catchment and percentage of rainfall delivered to the corresponding flume outlet during the study period.

	Total rainfall (mm)	Percentage of rainfall delivered to the catchment outlet
Flume 1	2085	71.90
Flume 2	2140	51.95
Flume 3	2030	59.05
Flume 4	2030	58.08
Flume 5	1897	64.65
Flume 6	1919	51.57
Flume 7	2247	51.84
Flume 8	2178	53.75
Flume 9	2061	42.60
Flume 10	2054	45.58
Flume 11	2178	42.28
Flume 12	2086	34.34
Flume 13	2025	41.15
Flume 14	2118	58.11
Flume 15	2138	77.38

#### 4.2.2 Water and sediment flux

For all flumes, a mean of 33.82% of the total water flux occurred during the rising limbs of storm events, 23.95% in the falling limbs and 42.4% during baseflow conditions (Table 3). A significantly higher proportion of sediment movement took place during the rising limbs of high flow events, with a mean of 62.87% for all flumes, compared with 19.00% in the falling limbs and 13.67% in baseflow conditions. It is therefore apparent that the ~2% of time spent in the rising limbs of storm events are most important for sediment erosion and transport to the edge-of-field flumes.

**Table 3:** Total fluxes of water and sediment during the study period (a) and the percentage of the fluxes occurring during each flow condition (b).

(a)	Water flux (m^3^)				Sediment flux (t)			
	Rising limb	Falling limb	Baseflow	Total	Rising limb	Falling limb	Baseflow	Total
Flume 1	1837	2181	3193	7211	725	504	253	1482
Flume 2	2553	2280	2559	7392	2456	681	559	3696
Flume 3	2928	2568	2439	7936	2939	584	687	4210
Flume 4	4399	4867	3845	13110	3321	796	675	4792
Flume 5	3131	2406	2483	8020	2316	500	701	3517
Flume 6	1141	710	1969	3820	604	194	121	919
Flume 7	1015	553	1460	3029	2025	474	459	2958
Flume 8	3384	2638	2196	8218	4045	891	1178	8245
Flume 9	2577	1933	2293	6804	1606	1029	224	2859
Flume 10	491	186	1027	1704	296	83	45	425
Flume 11	562	200	859	1621	347	78	55	480
Flume 12	535	159	581	1275	299	79	36	414
Flume 13	472	194	792	1458	435	117	111	663
Flume 14	724	359	1033	2117	912	311	225	1840
Flume 15	596	900	1052	2548	914	247	203	1696
(b)	Flow (%)			Sediment flux (%)				
	Rising Limb	Falling Limb	Baseflow	Rising Limb		Falling Limb	Baseflow	
Flume 1	25.47	30.25	44.28	48.92		34.01	17.10	
Flume 2	34.54	30.85	34.62	66.45		18.43	15.13	
Flume 3	36.90	32.36	30.74	69.81		13.88	16.32	
Flume 4	33.55	37.12	29.33	69.30		16.62	14.09	
Flume 5	39.04	30.00	30.96	65.84		14.23	19.92	
Flume 6	29.88	18.57	51.55	65.74		21.16	13.11	
Flume 7	33.52	18.27	48.21	68.46		16.04	15.51	
Flume 8	41.18	32.10	26.72	49.06		10.81	14.29	
Flume 9	37.88	28.41	33.70	56.17		35.99	7.83	
Flume 10	28.82	10.89	60.28	69.67		19.57	10.66	
Flume 11	34.64	12.36	52.97	72.38		16.18	11.47	
Flume 12	41.93	12.50	45.59	72.24		19.06	8.70	
Flume 13	32.36	13.33	54.33	65.66		17.63	16.77	
Flume 14	34.22	16.97	48.81	49.56		16.88	12.24	
Flume 15	23.38	35.31	41.30	53.87		14.56	11.94	

**Fig. 7:**
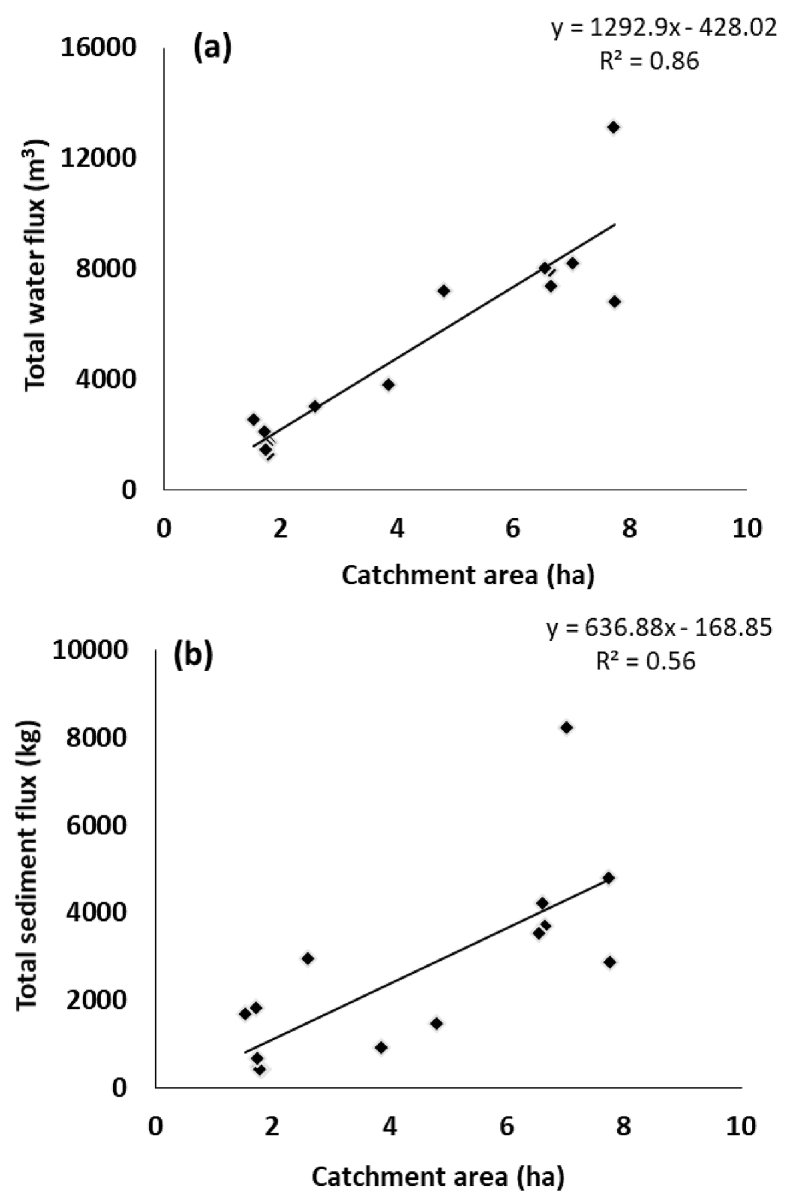
The relationships between total water (a) or sediment (b) flux and catchment area.

There was a strong linear relationship between the area of each catchment and its total water flux with an r^2^ of 0.85 (Fig. 7a). The relationship was found to be strongest (r^2^ of 0.90) when the catchment area is plotted against water flux in the rising limb only, compared to either the falling limb (r^2^ of 0.78) only, or baseflow (r^2^ of 0.75) only, reflecting the strong clockwise sediment hysteresis patterns observed. The total sediment flux was found to be more weakly correlated with catchment area with an r^2^ of 0.56 (Fig. 7b), most likely reflecting the effect of soil erodibility which was primarily impacted by ploughing. For the rising limb only, the corresponding r^2^ was 0.64, compared with 0.81 for the falling limb or 0.53 for baseflow only. The high r^2^ for the falling limb is likely due to its longer duration in larger catchments. Flume 8 is the outlier in these relationships with a high sediment flux for its area. This field was ploughed on the 06/07/2013 and subsequently re-seeded, providing a likely explanation for this result. When this flume and Flumes 14 and 15, which were also ploughed and had high SSC for their area, were removed from this part of the analysis, the r^2^ for the relationship between total sediment flux and catchment area increased to 0.73.

**Fig. 8:**
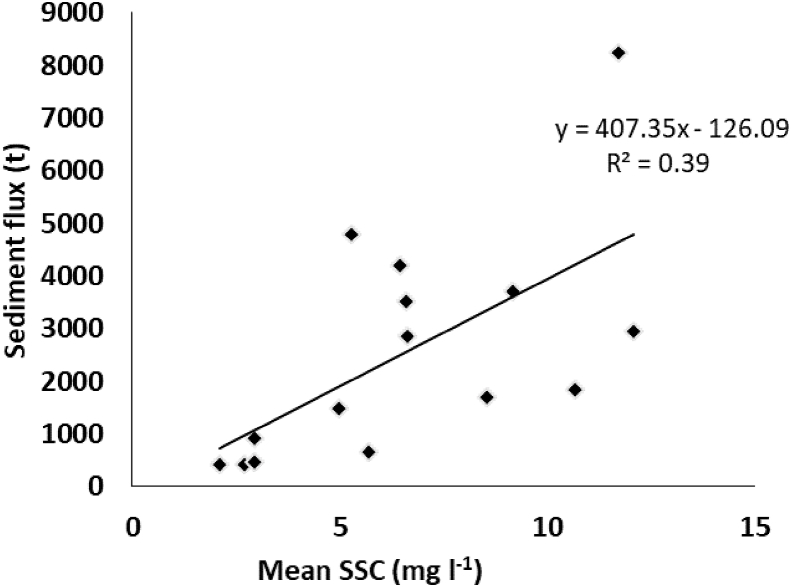
The relationship between the total sediment flux and mean SSC for each flume.

When comparing the total sediment flux to the mean SSC of runoff sampled in each flume (Supplementary Table 2), the relationship was weaker (r^2^ of 0.39) than that with catchment area or water flux (Fig. 8). The highest mean SSCs were recorded in Flumes 2, 7, 8, 14 and 15 which when combined were found to have an overall mean of 10.43 mg l^−1^ compared to a corresponding mean of 4.62 mg l^−1^ for the remaining flumes (Supplementary Table 2). Of these flumes, all but Flume 7 were ploughed in mid-2013.

### 4.3 Water and sediment yields normalised to catchment area and partitioned by flow

The water and sediment fluxes were normalised to catchment area and the duration of each flow condition (rising limb, falling limb and baseflow) to calculate partitioned water and sediment yields. Water yields ranged from approximately 4000 to 10000 m^3^ ha^−1^ yr^−1^ (Table 4). Flumes 1, 4 and 15 had the highest total specific water yields and Flume 12 the lowest. This flume was also found to have lowest percentage of rainfall delivered to the flume outlet (Table 2).

**Table 4:** Total specific water and sediment yields for each flume, partitioned by flow.

	Water yield (m^3^ ha^−1^ yr^−1^)				Sediment yield (t ha^−1^ yr^−1^)			
	Rising limb	Falling limb	Baseflow	Total	Rising limb	Falling limb	Baseflow	Total
Flume 1	63631	97797	3683	9000	3.74	1.61	0.03	0.18
Flume 2	60877	75888	2175	6673	5.44	3.92	0.03	0.33
Flume 3	71965	78816	2225	7197	9.04	1.49	0.06	0.38
Flume 4	68905	56980	3786	10155	6.83	0.89	0.05	0.37
Flume 5	79126	87403	2149	7362	7.25	1.43	0.06	0.32
Flume 6	89018	204381	2120	5941	5.36	2.78	0.02	0.14
Flume 7	118911	305943	2186	6994	27.43	12.99	0.09	0.68
Flume 8	66857	80144	1978	7028	14.59	3.02	0.11	0.71
Flume 9	62482	89228	1515	5271	5.95	1.62	0.03	0.22
Flume 10	160332	564717	1881	5621	9.86	7.47	0.01	0.14
Flume 11	166578	624639	1465	5529	10.65	7.80	0.02	0.16
Flume 12	150212	635945	1299	4300	10.76	10.79	0.01	0.14
Flume 13	173797	551349	1095	5002	15.10	10.73	0.03	0.23
Flume 14	159042	428680	1848	7389	26.92	20.36	0.07	0.64
Flume 15	90011	50321	4535	9933	18.21	1.78	0.07	0.66

Total specific sediment yields varied between 0.14 and 0.71 t ha^−1^ yr^−1^ with a mean of 0.36 t ha^−1^ yr^−1^ for all flumes. Specific sediment yields were far higher in the rising limbs (3.74 t ha^−1^ yr^−1^ – 27.43 t ha^−1^ yr^−1^) of storm events than in the falling limbs (0.89 t ha^−1^ yr^−1^ – 20.36 t ha^−1^ yr^−1^) and were very low during baseflow conditions (0.01–0.11 t ha^−1^ yr^−1^). The highest total specific sediment yields originated from Flumes 7, 8, 14 and 15. Of these, Flumes 8, 14 and 15 were ploughed and re-seeded in the summer of 2013. Flume 2 was also ploughed but had a sediment yield slightly lower than the mean for the entire dataset. Flume 7 had a high sediment yield but was not ploughed; this flume did, however, have the highest rainfall in the dataset with 8% more than the mean for all flume catchments. Flume 7 also has a notably finer particle size distribution than the other flumes with only 0.94% of the sample mass coarser than 250 μm compared to an average of 29.83% for the other flumes (Supplementary Fig. 6).

**Fig. 9:**
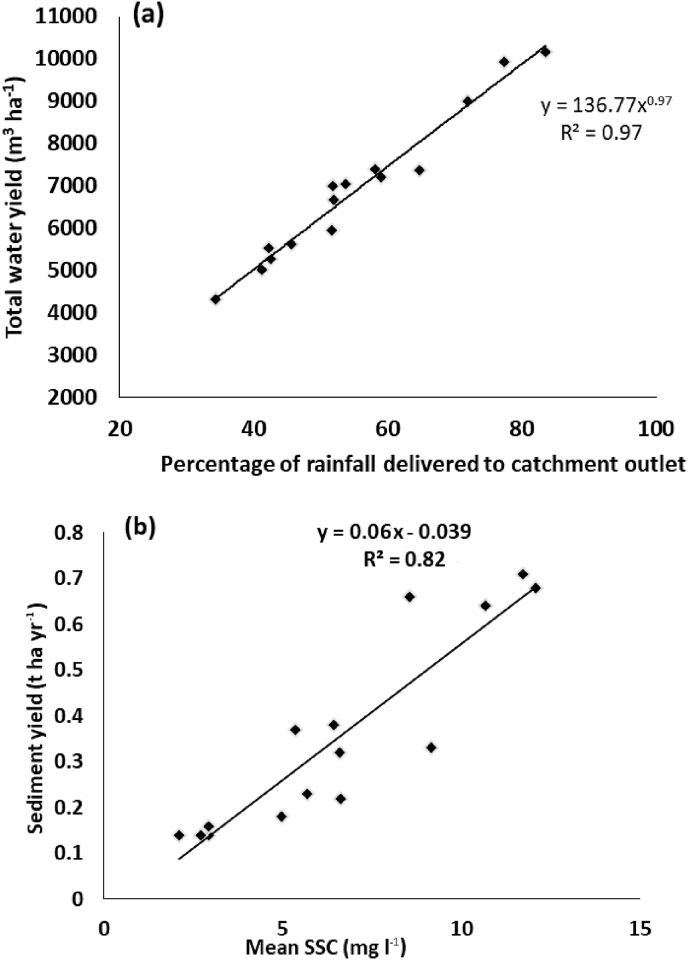
The relationships between the total water yield and percentage of rainfall delivered to the catchment outlets (a) and the total specific sediment yield and mean SSC sampled for the flume catchments (b).

The percentage of rainfall delivered to the catchment outlet (Table 2) was found to be the most strongly correlated factor with total specific water yield. This suggests that when catchment area is removed as a variable, it is the capacity of the fields to generate runoff that is the dominant controlling factor (Fig. 9a). There was found to be a spatial trend in the percentage of rainfall delivered to the catchment outlet (Supplementary Fig. 7), with Flumes 1 and 15 in the north having the highest delivery and Flumes 9, 10, 11,12 and 13 in the centre east having the lowest. This trend appears unrelated to catchment soil type or mean slope.

There was a strong relationship (r^2^ = 0.82) between total specific sediment yield and the mean SSC sampled for each flume (Fig. 9b). This relationship was much stronger than was found when examining the sediment flux. Sediment yield was also weakly correlated with both rainfall (r^2^ = 0.27) and water yield (r^2^ = 0.32). Therefore, when the catchment area is removed as a variable, the total specific sediment yield is primarily controlled by the erodibility of the catchment soils rather than the quantities of runoff reaching the flumes.

A Pearson correlation analysis of all variables was used to summarise the relationships between all of the potential controls and factors examined (Table 5). Catchment area was significantly correlated with all flow characteristics apart from the percentage of rainfall reaching the catchment outlet, indicating that area is the major component controlling water and sediment fluxes. Specific sediment yield was correlated with the mean SSC of the runoff, and weakly correlated with sediment flux and water yield.

**Table 5:** Pearson correlation coefficients of catchment, flow and sediment variables, values in bold are significantly correlated (p < 0.05).

	Rainfall	Percent time with animals	Rising limb water flux	Falling limb water flux	Baseflow water flux	Total water flux	Percentage time rising	Percentage time falling	Rainfall reaching outlet	Damaged soil area	Percent of soil area damaged	Water yield	Maximum flow rate	Mean SSC	Sediment flux	Sediment yield	Mean slope	Max flow accumulation
Area	−0.26	−0.25	**0.951**	**0.882**	**0.865**	**0.925**	**0.86**	**0.549**	0.329	**0.683**	**0.611**	0.117	**0.963**	0.202	**0.751**	0.031	0.244	**0.549**
Rainfall		0.225	−0.24	−0.21	−0.28	−0.24	−0.11	−0.06	−0.12	−0.23	−0.21	0.143	−0.27	**0.522**	0.156	**0.524**	−**0.52**	0.006
Percent of time with animals			−0.14	−0.17	−0.33	−0.21	−0.09	−0.10	−0.13	**0.641**	**0.692**	−0.06	−0.17	0.192	−0.27	−0.166	−0.08	−0.283
Rising limb water flux				**0.962**	**0.879**	**0.977**	**0.918**	**0.649**	0.489	**0.657**	**0.547**	0.21	**0.963**	0.242	**0.821**	0.159	0.286	**0.520**
Falling limb water flux					**0.924**	**0.991**	**0.93**	**0.783**	0.652	**0.657**	**0.522**	0.341	**0.883**	0.182	**0.733**	0.144	0.215	0.509
Baseflow water flux						**0.952**	**0.848**	**0.677**	**0.636**	**0.574**	0.457	0.374	**0.798**	0.109	**0.58**	−0.01	0.169	0.488
Total water flux							**0.926**	**0.724**	**0.604**	**0.651**	**0.527**	0.311	**0.911**	0.189	**0.742**	0.112	0.234	**0.520**
Percentage time rising								**0.853**	**0.700**	**0.539**	0.444	**0.535**	**0.857**	0.41	**0.824**	0.379	0.053	0.494
Percentage time falling									**0.863**	0.432	0.323	**0.71**	**0.549**	0.246	**0.521**	0.345	−0.06	0.320
Rainfall reaching outlet										0.243	0.131	**0.842**	0.327	0.252	0.347	0.457	−0.12	0.166
Damaged soil area											**0.968**	−0.05	**0.697**	−0.05	0.373	−0.07	0.222	**0.617**
Percent of soil area damaged												−0.06	**0.617**	−0.04	0.315	−0.07	0.142	**0.671**
Water yield													0.066	0.463	0.277	**0.564**	−0.45	0.089
Maximum flow rate														0.217	**0.773**	0.10	0.366	**0.574**
Mean SSC															**0.625**	**0.907**	−**0.53**	0.189
Sediment flux																**0.579**	−0.03	0.407
Sediment yield																	−0.48	0.079
Mean slope																		−0.052

The percentage of time animals were present in each catchment was found to be correlated with the area of soil damaged by poaching and the percentage of the total catchment area which is damaged (Table 5). However, these factors were not significantly correlated with either sediment flux or yield. Mean catchment slope was found to be unrelated to all variables apart from weak negative relationships with rainfall and mean SSC (Table 5).

## 5 Discussion

Three primary geographical components control water and sediment generation from the 15 study catchments monitored on the NWFP. First, and most importantly, catchment area is highly correlated (Table 5) with several response characteristics, namely: total water flux (r = 0.93) and its associated partitioning (r = 0.87–0.95), maximum flow rate (r = 0.96), maximum flow accumulation (r = 0.55) and sediment flux (r = 0.75). The primary effect of area is that larger catchments have more land which can release water and sediment, with the increased flow being of more minor importance with regards to sediment generation than drainage area.

Secondly, the mean SSC of the runoff sampled for each flume and catchment specific sediment yield are strongly correlated (r = 0.91; Table 5). SSC is also significantly correlated with sediment flux (r = 0.63; Table 5); however, this is a weaker relationship than with sediment yield. SSC is controlled by the capacity of each unit area of the hydrologically-isolated catchments to generate sediment. Most importantly, ploughed and re-seeded catchments generated a significantly higher mean SSC than undisturbed long-term permanent pasture catchments. Equally importantly, a higher SSC was generated for a given flow when soils are saturated compared to when dry. It is noteworthy that SSC was not strongly correlated with catchment area (r = 0.20; Table 5) suggesting that the greater runoff, slope length and flow accumulation from the larger catchments is not causing a proportional increase in SSC as might happen if concentrated flows were initiating rill or gully erosion rather than lateral wash.

Thirdly, total rainfall was found to be correlated, albeit relatively weakly (r = 0.52, Table 5) with sediment yield, indicating that the amount of net erosion taking place in a given area increases with rainfall. Field walking during storm events suggested that the majority of sediment was eroded and transported during active rainfall with such processes ceasing rapidly with the end of precipitation inputs. The percentage of rainfall reaching each catchment outlet was correlated with water flux and specific yield but was not correlated with sediment flux nor yield, indicating that these particular aspects of hydrological response are unlikely to be key controlling factors on the amount of erosion and sediment export taking place. Recent work has reported some statistically significant shifts in rainfall patterns for some sites with long-term records elsewhere in the UK with aspects of these changes involving higher mean rain totals on rain days and more back-to-back days delivering in excess of 30 mm of precipitation (Burt et al., 2016). Although such analysis was not repeated for the south west of England in conjunction with the work reported here, the relatively strong correlation (r = 0.52; Table 5) between rainfall and sediment yield suggests that such changes would potentially be significant for increasing sediment loss and associated on-site and off-site consequences in environmental settings similar to that represented by the NWFP. Here, it is noteworthy that recent papers have also reported the growing likelihood of weather extremes across the UK, including the unprecedented risk of higher rainfall (Thompson et al., 2017) and the importance of large-scale atmospheric-ocean oscillations (Mellander et al., 2018).

The finding that catchment area is the key control on sediment flux has the implication that either the entire area of each hydrologically-isolated catchment is eroding or that the greater catchment area is resulting in a larger quantity of runoff which is concentrating into high velocity flows which, in turn, cause more erosion. A higher flow accumulation in each catchment was shown to increase sediment fluxes and yields; however, these correlations were weak (Table 5). Additionally, Flume 2 was ploughed and flow accumulation was more concentrated than in the unploughed Flume 7, yet it did not have a higher sediment yield. Instead, Flume 7 with the higher rainfall (Table 2) experienced the higher total specific sediment yield (Table 4). As catchment area is more strongly correlated with sediment flux than maximum flow accumulation (Table 5) it is likely that erosion is occurring across the entire catchment areas. This is in contrast to the findings of Parsons et al. (2006) who found that sediment yield decreased with increasing plot length once a threshold of 7m length is passed because of the limited travel distance of individual particles.

It was found that SSC decreased sharply and rapidly when rainfall stopped, indicating that the action of rainfall is eroding the sediment subsequently reaching the edge-of-field flumes. It was also found that a thin layer of surface runoff covered almost the entire field areas during the latter stages of storm events. It is therefore proposed, on the basis of the analysis of the monitoring data herein and associated field observations, that raindrop impact is the primary agent of soil detachment and that saturation-excess surface runoff transports the detached particles to the edge-of-field where they are intercepted by the network of French drains. During the latter stages of a high-intensity storm event on the 26th of November 2018 it was observed that the turbidity of runoff decreased significantly after rainfall stopped, confirming the observations made using the turbidity records. It was also observed that concentrated high velocity flows over disturbed and trampled earth were insufficient to entrain soil particles (Supplementary Fig. 8). It is possible that during the early stages of storm events there is a greater concentration of easily detached particles and this erosion mechanism is of importance. However, no significant detachment of particles by concentrated overland flows have yet been observed on the site, even on heavily trampled soil.

This mechanistic conceptualisation centred on raindrop-impacted saturation-excess overland flow erosion is supported by the findings of previous work. Raindrop impact and splash have been identified as important components of rain-induced soil erosion (Zhang et al. 2017b, 2019; Hao et al., 2019). Here, the impact of the raindrops is responsible for two important processes increasing the propensity for soil erosion; topsoil aggregate breakdown and instigation of soil fragment movement (Legout et al., 2005; Warrington et al., 2009). Several studies have reported that raindrop impact can break down soil aggregates to help initiate soil erosion (Ekern, 1951; Kinnell, 1990a, 1990b; Van Dijk et al., 2002; Wang et al., 2014). Here, aggregate breakdown can be attributed to slaking, physico-chemical dispersion or differential clay swelling (Le Bissonnais, 1996; Levy et al., 2003). Whilst the observational work on the study site is not currently investigating or apportioning the principal mechanisms of aggregate breakdown experimentally, it is feasible to assume that raindrop impact is the first stage of soil erosion. A rapid decline in soil erosion in field settings where soil wetting is fast has been attributed to a dominant role of slaking in aggregate breakdown (Grant and Dexter, 1990; Ramos et al., 2003; Zaher and Caron, 2008), but targeted experimental work is clearly required at our study site to confirm or counter this, even though sediment responses were observed to be rapid. Regardless, published work has previously reported soil erosion by raindrop impact and subsequent transportation by raindrop-impacted overland flow (Young and Wiersma, 1973; Meyer et al., 1975; Kinnell, 2005). Similarly, previously reported studies have underscored that sediment transport by overland flow is greatly enhanced by raindrop impact (Foster, 1982; Singer and Walker, 1983; Guy et al., 1987) and that raindrop-impacted overland flow soil erosion is a detachment-limited process (Lattanzi et al., 1974; Meyer et al., 1975). Again, these findings point to our conceptualisation of soil erosion at the study site on the basis of sediment monitoring and field observations. This conceptualisation is reinforced by the fact that soil moisture was identified as a key control on temporal trends in sediment flux, with rainfall during the dry summer period of 2013 resulting in little flow and sediment flux. Even when soil moisture had recovered close to its pre-summer levels later in 2013, sediment flux continued to be significantly lower than when the soil was fully saturated (Fig. 4). It has been identified in catchments elsewhere globally that raindrop impact is the primary mechanism for soil detachment where overland flows are the dominant transport mechanism (Ellison, 1945; De Ploey and Savat, 1968; Young and Wiersma, 1973; Moss et al., 1979; Parsons et al., 1994).

This work identified a rapid and sharp decline in erosion after rainfall ceases. It has been identified (Ellison, 1945) that the reduced availability of loose detachable soil particles in the latter stages of a storm event can also cause the same reduction of sediment transport in the falling limbs of events as observed in this study. The cultivation of fields clearly has the effect of removing the protective grass sward and breaking up the soil structure, thereby increasing the quantity of loose detachable particles prone to mobilisation and delivery in saturation-excess overland flow in the catchments. Here, a reduction in erosion in the latter stages of high flow events may also be linked to the presence of the surface water. A thin layer of water up to that of the raindrop diameter has been shown to increase erosion because of turbulence in the water film (Palmer, 1963, 1965), but deeper surface water reduces erosion by providing a protective barrier against more detachment by raindrops, therefore resulting in eventual exhaustion of the supply of readily mobilised soil particles (Dunne et al., 2010). This links well with the soil particle detachment-limitation noted above.

Mean catchment slope was weakly negatively correlated with total rainfall and mean SSC (Table 5) suggesting that slope also plays only a minor role in the generation of sediment from the NWFP. In much published research, slope has been shown to have an important effect on rain splash erosion with dislodged particles being preferentially pushed in a down-slope direction (Dunne and Dietrich, 1980). Froehlich and Slupik (1980) and McCarthy (1980) showed that steeper slopes were characterised by a significant increase in soil detachment by rain splash. These studies were, however, over a range of gradients between 0°–25° and 0°–20° and it is therefore possible that the range of mean gradients for the hydrologically-isolated catchments on the NWFP (4.17°–12.25°; Table 1) is insufficient to show an effect in the analysis reported herein. It has also been shown that other factors such as raindrop size, vegetation cover and soil mobility have a larger effect than slope gradient (Dunne et al., 2010). Equally, other field-based studies in the UK have detected little relation between slope and the severity of soil erosion (Morgan, 1977; Evans, 1990b; Evans and Brazier, 2005), raising concerns about the prominent role slope plays in the computations by many erosion models.

An increased percentage of time with livestock present in each catchment resulted in a larger area of damaged soil. Larger catchments also had larger areas of damaged soil, possibly due to the livestock congregating in a small area of the field e.g. in conjunction with the regular moving of feeder rings. Neither the total area or percentage of the catchment area with damaged soil were, however, correlated significantly with sediment flux or yield (Table 5). It is possible that the small effect of ruminant livestock related soil damage is masked by the large impacts of the ploughing and re-seed in some catchments. It is also noteworthy that even in the most damaged catchment, only 4.1% of the total field area was bare and damaged by poaching during the study period, again limiting the impact of such features of the pastures. This specific finding is potentially important for the management of soil loss from lowland grazing more generally in the UK, since the targeting of on-farm mitigation measures for erosion control has frequently focussed on measures such as regular movement of feeder rings before excessive trampling damage occurs, installation of concrete bases to protect soils beneath and surrounding drinking troughs, re-siting gateways away from high risk areas and the resurfacing of heavily poached gateways and cattle tracks (Collins et al., 2016). The findings from the study herein, however, suggest that such mitigation is unlikely to deliver substantial benefits for erosion management, highlighting the importance of using high resolution data to develop mechanistic (e.g. hydrological) understanding for guiding management interventions, rather than being informed by purely visual evidence alone. In this case, the monitoring data and analysis suggest that more general grazing management (e.g. reducing field stocking rates when soils are wet) will be more important (Kemp and Michalk, 2007), although since the NWFP aims to follow best practice, the scope for significant changes to the stocking density and grazing regime is small, especially in the context of the need for productive agriculture.

## 6 Conclusions

This research highlights the importance of particle detachment by raindrop impact and saturation-excess surface runoff for sediment mobilisation and delivery. The significant control imparted by catchment area suggests that connectivity within the studied fields is extremely high. This is likely due to widespread saturation-excess overland flow as driven by the local soils, although the network of French drains installed for the hydrological-isolation is a factor in the connectivity between the fields and flumes.

This study was conducted in an area of the UK for which it has recently been reported that a scenario of future projected uptake (rate = 95%) of on-farm mitigation measures might feasibly result in a reduction in sediment delivery to river channels from agricultural land by 39%. The modelled work suggested that much of this reduction could likely be achieved through targeted source control rather than delivery control (Zhang et al., 2017a). The findings reported in this paper support X.C. Zhang et al. (2017b) as the ploughing and re-seeding of some of the catchments resulted in a significant increase in sediment generation due to the entire field areas being exposed to raindrop impact and sediment transport by saturation-excess runoff. However, further research is needed into the effects of ploughing and re-seeding in lowland grazing systems on erosion rates and processes. One catchment on the NWFP had an extremely strong response to ploughing whilst another experienced very little increase in sediment flux. Understanding the geographical factors controlling these differences observed for fields in the same locality is key for reducing soil erosion and excess sediment loads exported to aquatic environments in lowland grazing landscapes. The ongoing work on the NWFP will provide the opportunity to answer these questions on the basis of mechanistic understanding provided by a combination of high resolution quasi-continuous monitoring and information for both intrinsic and management factors.
