# Supplementary figures and images for: Field-based determination of controls on runoff and fine sediment generation from lowland grazing livestock fields
Source: J Environ Manage. 2019 Nov 1;249:109365. doi: 10.1016/j.jenvman.2019.109365 (PMC6876281; doi:10.1016/j.jenvman.2019.109365)

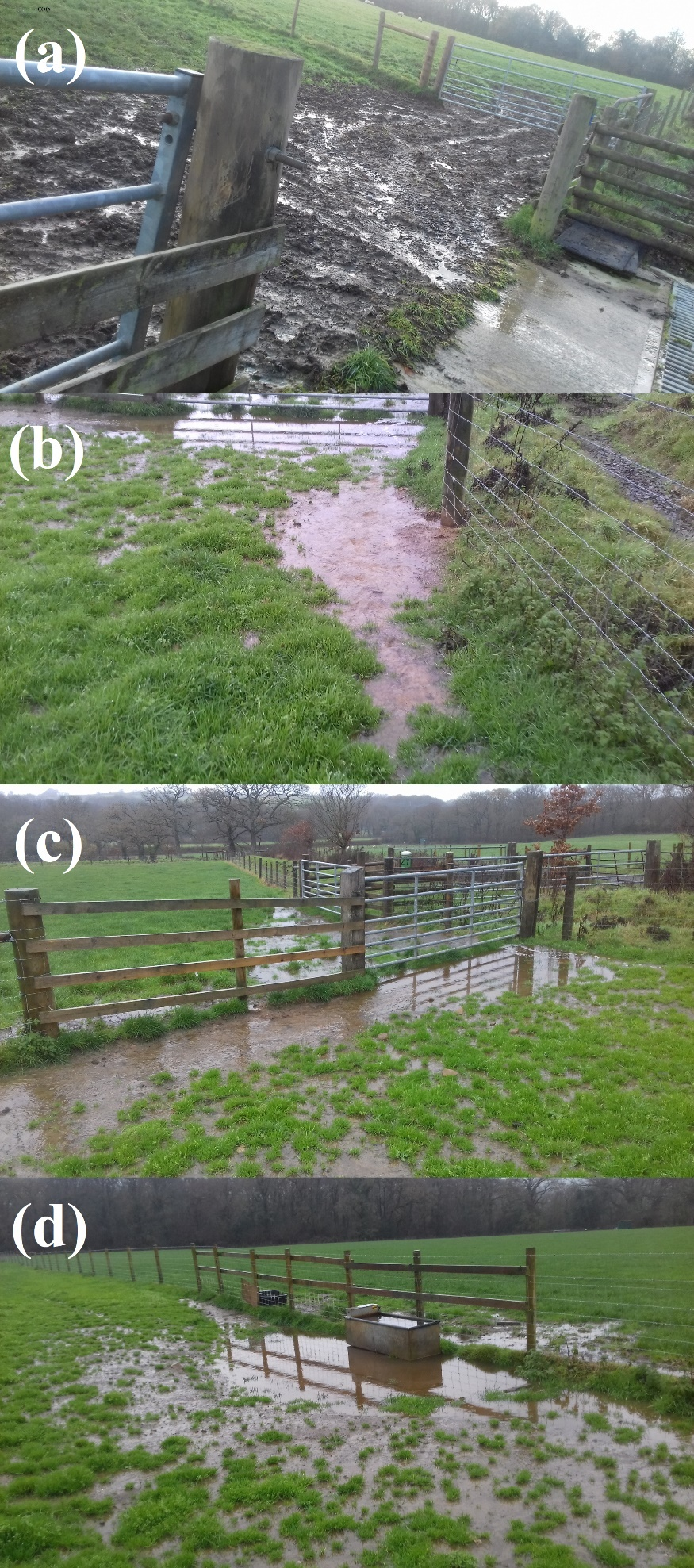

Supplement: Multimedia component 1 [file mmc1.zip › Supplementary Figure 8.tif]

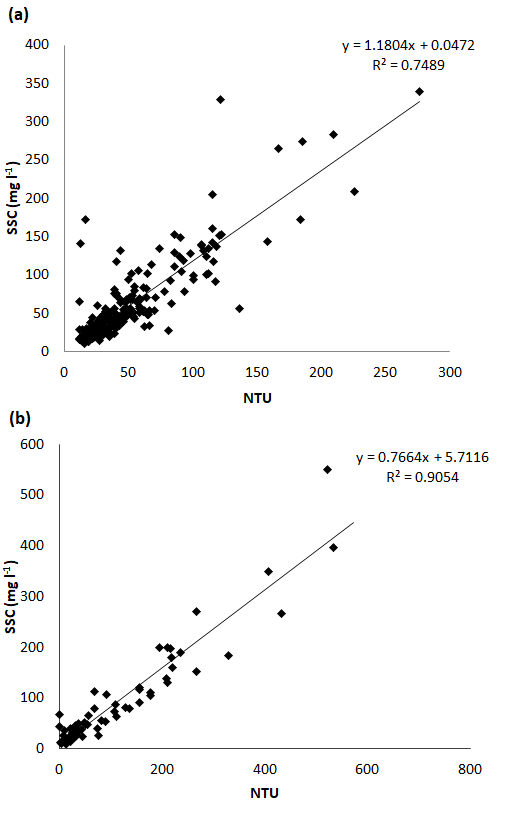

Supplement: Multimedia component 1 [file mmc1.zip › Supplementary Figure 1.tif]

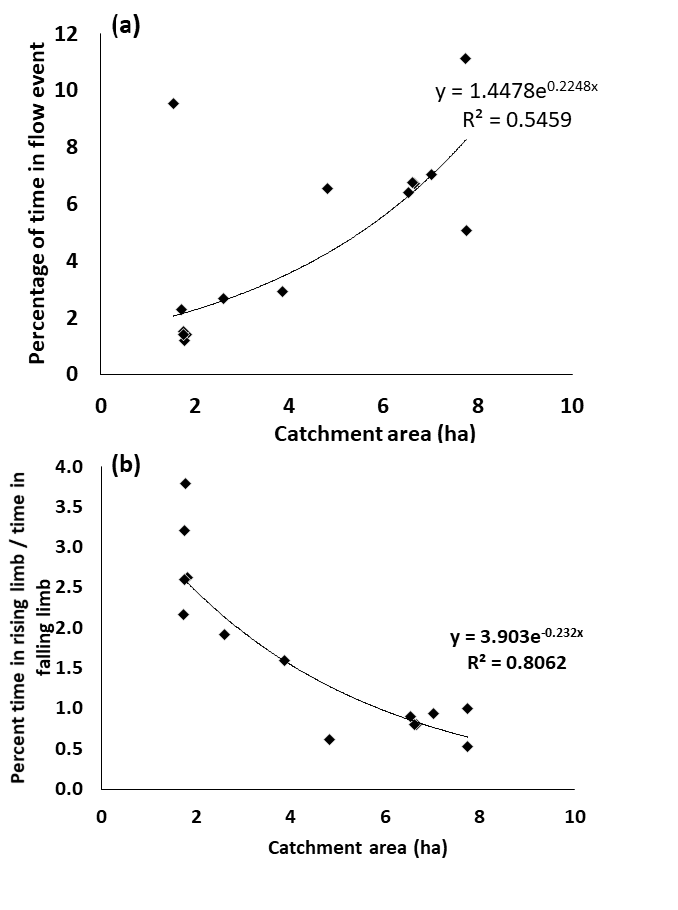

Supplement: Multimedia component 1 [file mmc1.zip › Supplementary Figure 2.tif]

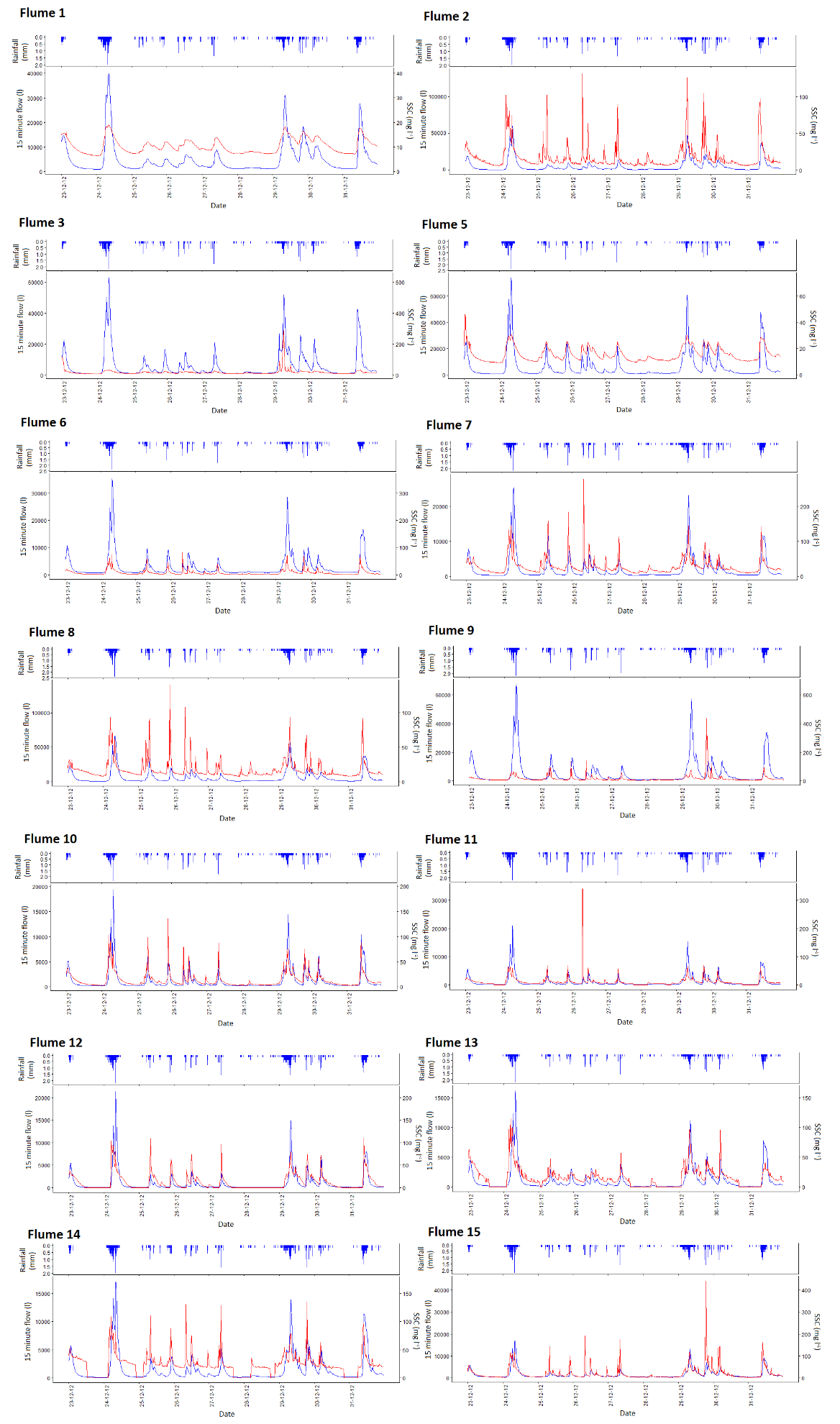

Supplement: Multimedia component 1 [file mmc1.zip › Supplementary Figure 3.tif]

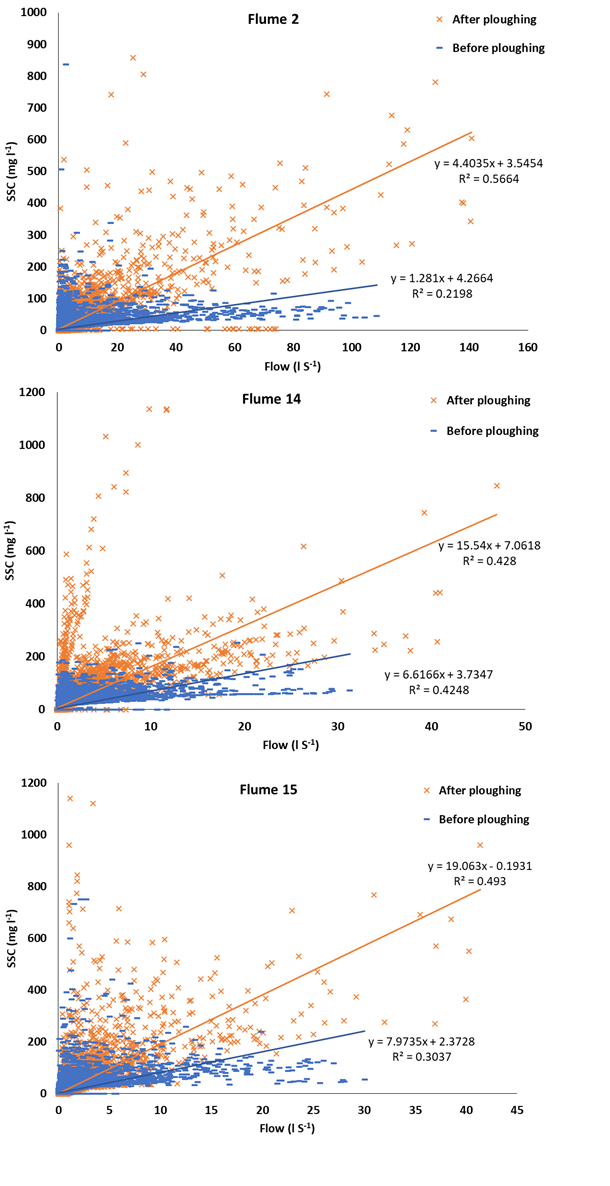

Supplement: Multimedia component 1 [file mmc1.zip › Supplementary Figure 4.tif]

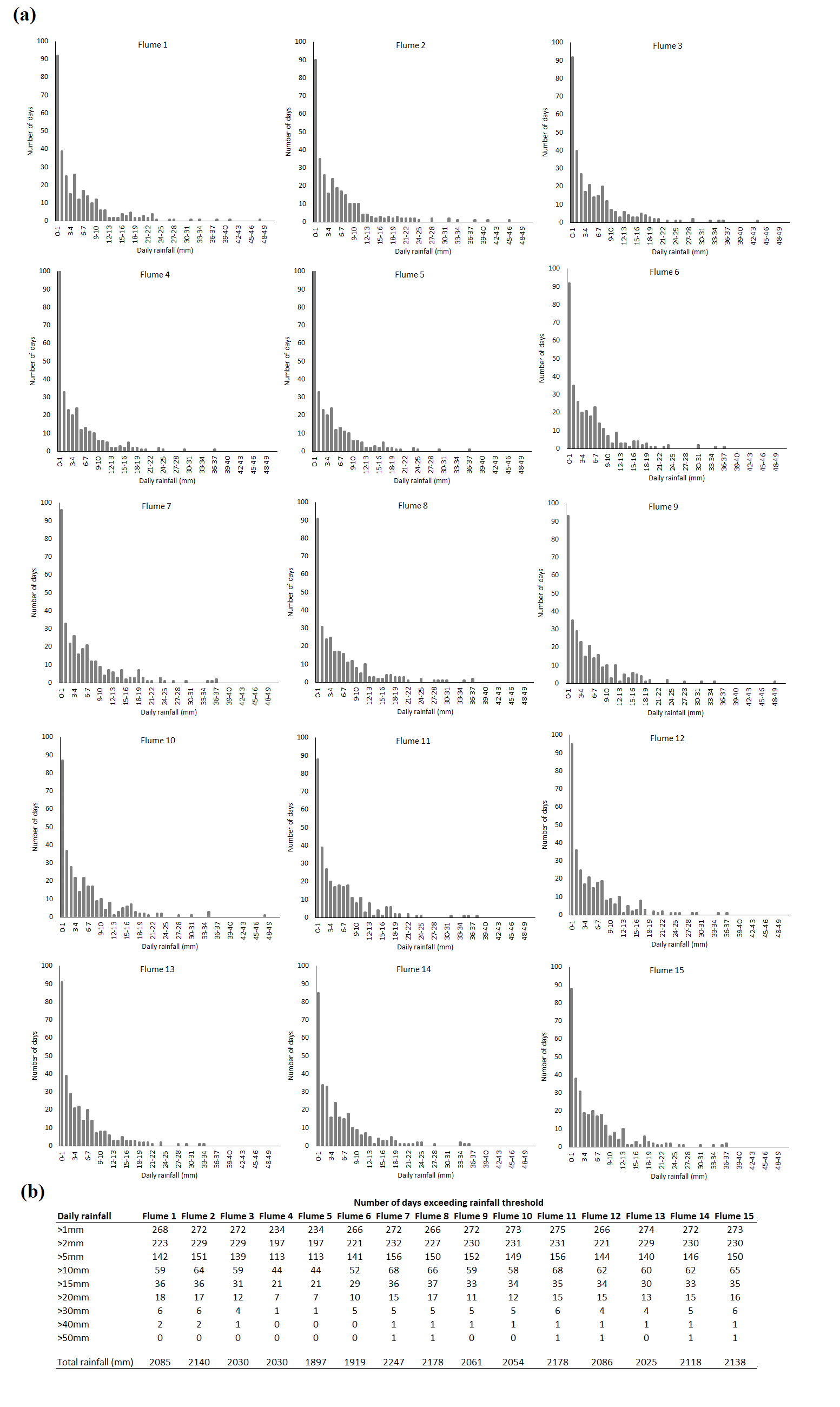

Supplement: Multimedia component 1 [file mmc1.zip › Supplementary Figure 5.tif]

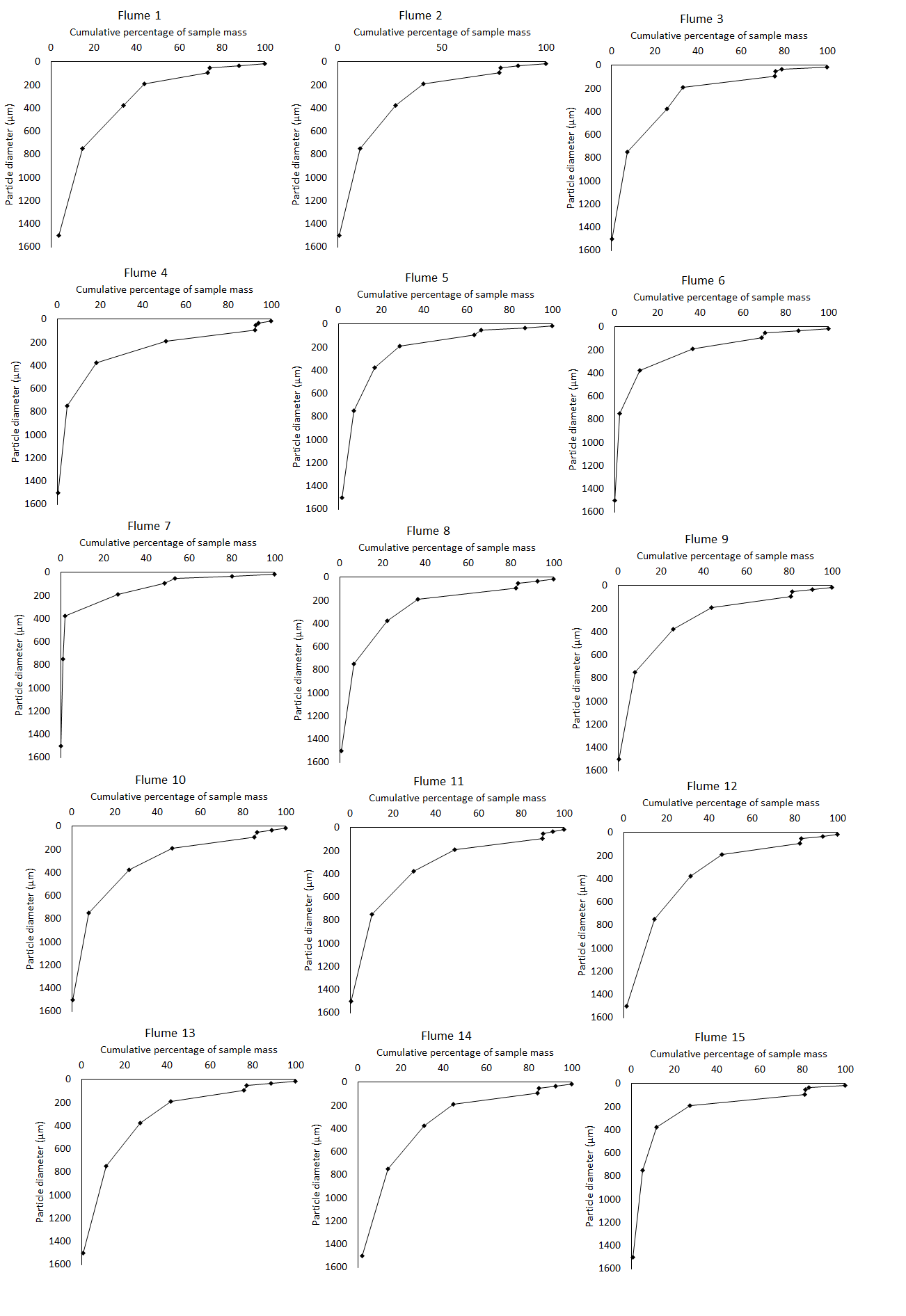

Supplement: Multimedia component 1 [file mmc1.zip › Supplementary Figure 6.tif]

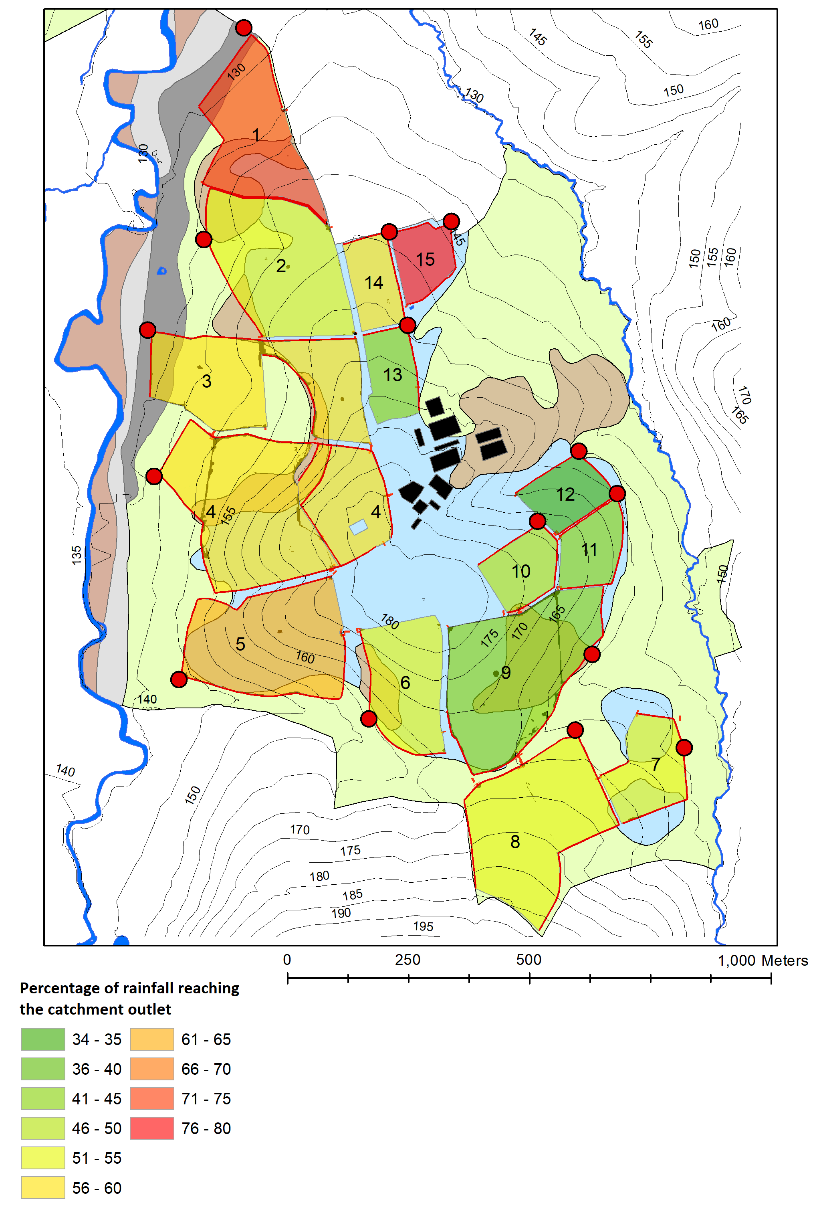

Supplement: Multimedia component 1 [file mmc1.zip › Supplementary Figure 7.tif]
