# Supplementary material for: Field-based determination of controls on runoff and fine sediment generation from lowland grazing livestock fields
Source: J Environ Manage. 2019 Nov 1;249:109365. doi: 10.1016/j.jenvman.2019.109365 (PMC6876281; doi:10.1016/j.jenvman.2019.109365)
Supplement: Multimedia component 2 [file mmc2.docx]

Supplementary Table 1. The percentage of time in each flow category for each flume.

|  | Rising limb | Falling limb | baseflow |
| --- | --- | --- | --- |
| Flume 1 | 2.48 | 4.07 | 93.45 |
| Flume 2 | 2.98 | 3.73 | 93.29 |
| Flume 3 | 3.01 | 3.75 | 93.24 |
| Flume 4 | 3.86 | 7.25 | 88.89 |
| Flume 5 | 3.04 | 3.37 | 93.59 |
| Flume 6 | 1.79 | 1.12 | 97.08 |
| Flume 7 | 1.75 | 0.91 | 97.34 |
| Flume 8 | 3.41 | 3.63 | 92.96 |
| Flume 9 | 2.52 | 2.53 | 94.95 |
| Flume 10 | 1.01 | 0.38 | 98.61 |
| Flume 11 | 1.14 | 0.35 | 98.51 |
| Flume 12 | 0.95 | 0.25 | 98.80 |
| Flume 13 | 1.02 | 0.39 | 98.59 |
| Flume 14 | 1.57 | 0.72 | 97.70 |
| Flume 15 | 2.58 | 6.97 | 90.45 |

Supplementary Table 2: The mean SSC of flow sampled for each flume during the study period.

| Flume | Flume 1 | Flume 2 | Flume 3 | Flume 4 | Flume 5 | Flume 6 | Flume 7 | Flume 8 |
| --- | --- | --- | --- | --- | --- | --- | --- | --- |
| Mean SSC (mg l^-1^) | 4.97 | 9.16 | 6.43 | 5.27 | 6.6 | 2.93 | 12.1 | 11.7 |
| Flume | Flume 9 | Flume 10 | Flume 11 | Flume 12 | Flume 13 | Flume 14 | Flume 15 |  |
| Mean SSC (mg l^-1^) | 6.62 | 2.7 | 2.93 | 2.09 | 5.69 | 10.7 | 8.55 |  |
